# Quantifying the mixing behavior of direct injected hydrogen in high-pressure environments by Rayleigh scattering

**DOI:** 10.1007/s00340-025-08549-1

**Published:** 2025-09-06

**Authors:** Max Peters, Noud Maes, Nico Dam, Jeroen van Oijen

**Affiliations:** Department of Mechanical Engineering, Eindhoven University of Technology, P.O. Box 513, 5600 MB Eindhoven, The Netherlands

## Abstract

In the framework of the Argon Power Cycle, millisecond-pulsed hydrogen gas injections into a high-pressure, room temperature nitrogen or argon ambient are investigated. Instantaneous Rayleigh scattering is used to quantify the hydrogen mole fraction in the ensuing jets. A readily available HDEV injector with a straight 0.55-mm orifice and an inward-moving needle controls the mass flow into the constant-volume setup in accordance with (compressible) choked flow theory. The linear dependence of the Rayleigh signal on the number density is experimentally validated and the validity for the assumed constant number density throughout the mixing jet is presented. The evolution of mole fraction is presented for both nitrogen and argon ambient gases, and pressure ratios of 2.5 and 10. Quasi-steady behavior is shown in both axial and radial direction, while self-similar behavior is already observed 3 mm from the nozzle ($x/d_\textrm{e} = 5.5$).

**Supplementary Information:**

The online version contains supplementary material available at 10.1007/s00340-025-08549-1.

## Introduction

Decarbonization of the power generation and transport sectors has become a necessity to keep climate change at bay. One of the carbon-free alternatives is green hydrogen, which can be formed by the electrolysis of water when there is a surplus of renewable (electrical) energy available. One of the most cost-effective and robust ways to liberate the stored energy is to use the hydrogen as a fuel for an internal combustion engine [1–5]. Some major challenges of hydrogen combustion are its low minimum ignition energy, and its high flame temperature that lead to the potential formation of nitrogen oxides ($\mathrm {NO_x}$) [6–8].

An Argon Power Cycle (APC) could solve these challenges, as the nitrogen working fluid is replaced by argon in the combustion chamber [9–11]. This opens the pathway to more efficient CI combustion, the efficiency of which is further elevated by the increased specific heat ratio ($\gamma$) of the monoatomic constituent in the working fluid. For such an engine, fuel and oxygen delivery to the combustion chamber is needed, introducing the need for high-pressure direct injection of hydrogen (and oxygen) into a pressurized (enclosed) environment.

In this paper we focus on high-pressure injections of hydrogen into various ambient conditions. Jet visualization (angle, penetration) and mass flow rate characterizations have been performed on hydrogen jets at suitable timescales for combustion engines in the past, mainly utilizing Schlieren (or Shadowgraphy) and pressure transducer measurements in constant-volume chambers at room temperature [11–16]. These studies did not address one major facet for CI combustion, typically showing mixing-controlled combustion behavior: how does the hydrogen jet resulting from high-pressure injection mix with the pressurized environment?

Quantification of the hydrogen mixing with the ambient gas is far less documented, with the main results recently provided by Wu et al.  who performed simultaneous high-speed Rayleigh and Raman imaging on a jet emanating from a hollow-cone injector [17]. This jet was evaluated at a distance of 40 mm downstream from the injector, assuming isothermal behavior. Alternatively, tracer doped laser-induced fluorescence is often employed in situations where reflections and Mie scattering cannot be easily contained, such as in optically-accessible combustion engines [18–28]. The fluorescence wavelength shift allows to discriminate the fluorescence from elastic scattering. However, depending on the used fuel, a suitable tracer with matching fluid properties needs to be selected, while the (collisional) quenching rate can be hard to determine based on the (local) ambient composition. Raman scattering is an appealing alternative, as both temperature (red versus blue shift) and composition can be determined simultaneously. However, the Raman scattering efficiency is very low, making transient measurements impossible in a tall laser light sheet. As these hydrogen jets are highly turbulent while moving through the optically accessible vessel on a millisecond timescale, and there is no tracer available with properties close to hydrogen, Rayleigh scattering is selected in this study, as the relatively high scattering efficiency enables single-shot visualizations to capture a substantial portion of a combustion engine-sized jet using a ns-pulsed Nd:YAG laser for illumination.

Rayleigh scattering has been employed in the past to image mole fraction distributions inside turbulent gaseous jets [29–32], while spectrally-resolved Rayleigh scattering measurements are possible to additionally determine velocity, temperature, and density fluctuations in high-speed flows [33]. Additionally, Rayleigh scattering was used for visualization of structure and mixing behavior of diesel sprays. The first employment of Rayleigh scattering on diesel sprays is reported by Espey et al. in 1997, who evaluated fuel concentrations inside an optically accessible engine, while the spray is evaporating and combusting [34]. Schulz et al. showed that quantitative fuel concentration measurements are possible inside their optically accessible diesel spray chamber [35]. Single-shot measurements on the evaporated portion of the engine-sized jet were reported by Idicheria et al., quantifying the fuel-oxidizer mixture inside the mixing spray inside a constant-volume vessel using an Engine Combustion Network (ECN) Spray A injector [36]. These results were further improved by Manin et al. employing improved image processing techniques and a pulse-burst laser system, allowing high-speed imaging [37]. Wan et al. applied these measurements for both ECN Spray A and D injectors, which represent passenger car and heavy-duty injection systems, respectively [38].

The novelty of experimentally quantifying the mixing field of a fully developed hydrogen jet by Rayleigh scattering is clear, but also very valuable regarding auto-igniting jets. The results could be particularly valuable for modeling, comparing the quasi-2D mole fraction fields to cold flow computational fluid dynamics simulations.

This paper is constructed as follows: The theoretical background and assumptions for the quantification are followed by the descriptions of the experimental setup, and post-processing methods in the methodology section. The results section discusses a validity check of some of the assumptions and presents the evolution of mole fraction field inside the jet throughout time. The jets are evaluated at different injection pressures and at two different pressure ratios for argon and nitrogen ambient gases. Quasi-steady behavior is shown both axially and radially throughout the jet, while self-similarity has been found throughout the measurable area. Lastly, conclusions are presented.

## Methodology

### Planar Rayleigh scattering

To visualize the mixing behavior of the highly turbulent hydrogen jets in argon and nitrogen environments, planar Rayleigh scattering (PRS) has been employed. PRS is a non-spectroscopic laser diagnostic that also enables the visualization and quantification of monoatomic species. As noble gases only have electronic states available, vibrational or rotational states cannot be explored. In addition, the relatively large Rayleigh scattering efficiency allows for single-shot measurements in pressurized environments.

If the position of the detector and the detection system remain identical between measurements, the solid angle of detection remains constant, and the photon count is proportional to the Rayleigh scattering intensity, the counts on a specific pixel ($I_\textrm{r}$) can be assumed as linearly proportional to the incident laser power $I_0$, number density (*N*) in the probed volume (*V*), and molecule dependent scattering cross-section ($\sigma _i$). When including the (possibly pixel-dependent) efficiency of the detection system ($\eta _\textrm{sys}$), the measured pixel values becomes [36, 39]:

1 $$\begin{aligned} I_\textrm{r} = \eta _\textrm{sys} I_0 N \sigma _i, \end{aligned}$$

with

2 $$\begin{aligned} \sigma _i = \frac{32\pi ^3}{3\lambda ^4} \left( \frac{n_i-1}{N_0}\right) ^2 \frac{3}{3-4\rho _v}, \end{aligned}$$

where $\lambda$ is the wavelength of the incident light, $n_i$ is the refractive index of the pure gas at STP, $N_0$ is the total molecular number density at STP and $\rho _v$ is the depolarization ratio of species *i*. The calculated $\sigma _i$ and respective values for the gases used in this work can be found in Table 1 [40–43].

**Table 1:** Calculated Rayleigh cross-section per species (at 532 nm) including refractive index and depolarization ratio [40–43]

Species	σ_i_	n_i_−1	ρ_v_
–	$\cdot 10^{27}$ $[{\text {cm}}^{2}]$	$\cdot 10^4$	$\cdot 10^2$
Hydrogen	1.13	1.395	0.98
Helium	0.0697	0.3493	–
Argon	4.56	2.8268	–
Nitrogen	5.23	3.002	1.12

Finally, the pixel values obtained in Rayleigh scattering of a mixture of different species can be calculated using the mole faction ($X_i$):

3 $$\begin{aligned} I_\textrm{r} = \eta _\textrm{sys} I_0 N \sum _i X_i \sigma _i. \end{aligned}$$

### Optical vessel and hydrogen injector

**Fig. 1:**
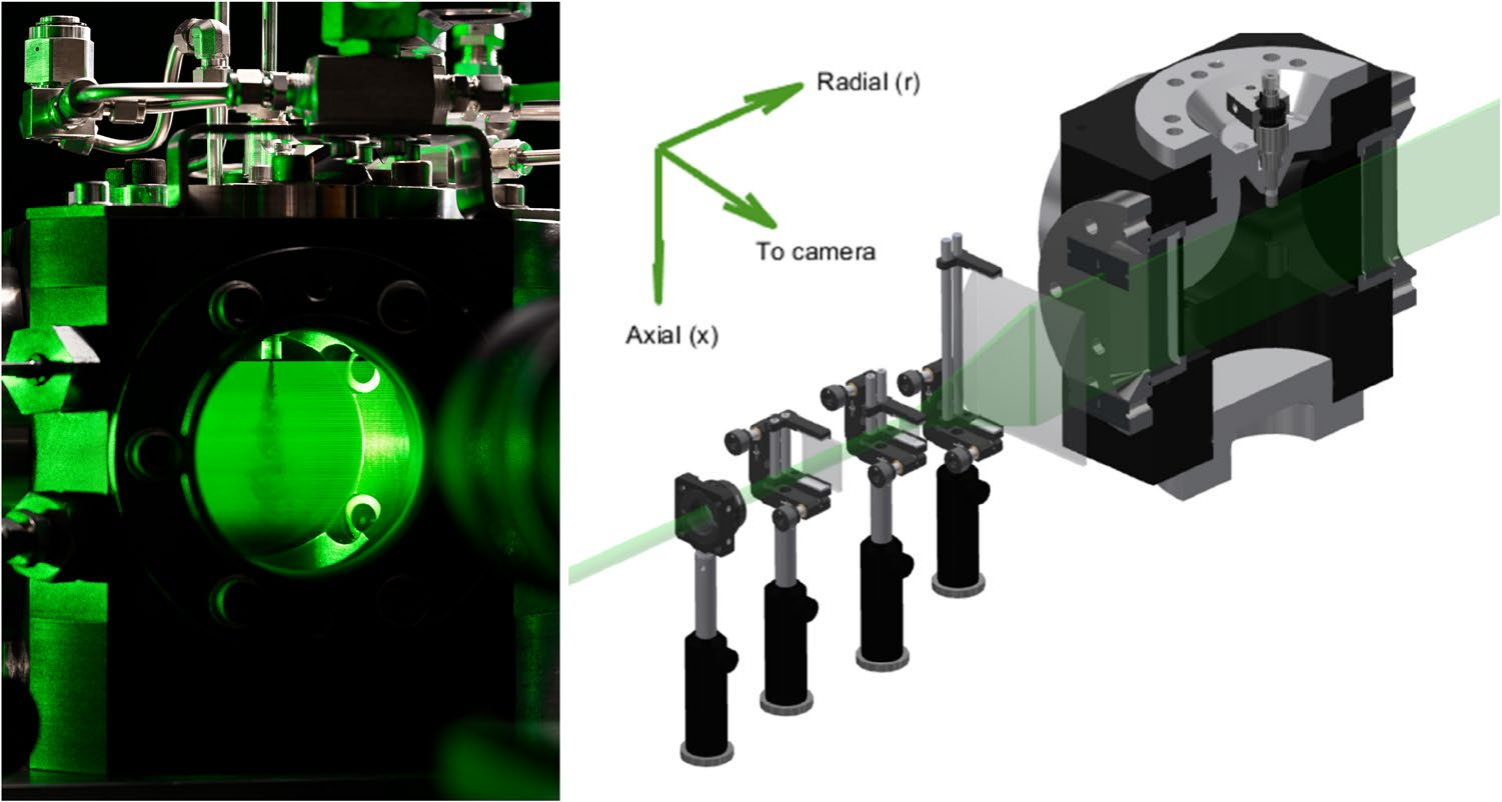
Left panel: Rayleigh scattering of a 10-MPa hydrogen jet in a 1-MPa argon ambient, 5 ms aSOA. The DSLR camera viewpoint is on the left of the scientific camera used for the measurements. The right panel shows an overview of the optical setup and the cross-sectional view of the optically accessible vessel with top-mounted injector

The high-pressure injection of hydrogen is conducted in a constant volume vessel at ambient temperature, shown in Fig. 1. The vessel is optically accessible through a 90-mm quartz window, while a laser sheet can enter and exit the vessel through anti-reflection-coated slit windows. This cylindrical chamber can be operated at an absolute ambient pressure ($p_{\textrm{a}}$) up to 40 bar. In this work we have used either argon or nitrogen as the ambient gas.

Hydrogen is injected through a HDEV 1.1 Gasoline Direct Injection (GDI) injector (OEM: 06F 906 036) with a single straight hole with diameter $d_\textrm{e}= {0.55\,\textrm{mm}}$. The nozzle is rated at a maximum fuel pressure ($p_\mathrm {{f}}$) of 10 MPa, while the injector solenoid allows for a maximum injection actuation duration ($t_\textrm{act}$) of 10 ms. Experimental timing is referenced to Start of Actuation (SOA) for accuracy, where $t= 0$ is the moment when the injector is electronically actuated. Start of injection (SOI) occurs at 0.2 ms after SOA (aSOA) and it takes approximately 0.1 ms for the nozzle to be fully open, which is not further mentioned from here on. The hydrogen mass flow ($\dot{m}=0.86\,\text {mg/ms}$), discharge coefficient ($C_\textrm{d} = 0.56$), and inner geometry were characterized in the same manner as in earlier work [16]. An STL format file containing the inner-nozzle geometry in open (needle) position is as supplementary material online available with this paper. From this earlier work using high-speed Schlieren imaging and injected-mass experiments, jet penetration was found to be governed by the nozzle Pressure Ratio (nPR) at room temperature, while the reservoir pressure ($p_\mathrm {{f}}$) at constant temperature independently sets the injected mass for choked flow conditions, when $\textrm{nPR} = \frac{p_\textrm{f}}{p_\textrm{a}}$ $> 1.899$ [16]. The chosen experimental conditions are presented in Table 2.

**Table 2:** Range of experimental conditions, chosen such that the choked flow limit applies ($\textrm{nPR} > 1.899$)

Experimental conditions				
nPR	–	10	10	2.5
$p_\textrm{f}$	MPa	10.0	4.0	4.0
$p_\textrm{a}$	MPa	1.0	0.4	1.6
$t_\textrm{act}$	ms	3	3	10
$n_\textrm{a}$	–	Nitrogen	Nitrogen & Argon	Argon
$T_\textrm{a}$	K	291		
$T_\textrm{f}$	K	291		

### Optical system

A back-lit CCD camera (Pixis 1024BR Excelon) captures the scattering perpendicular to the laser light sheet through a Nikkor 50-mm f/1.2 lens and 11-mm spacer, resulting in a projected pixel size of 0.087 mm/pixel. An Andover 532-nm HBW 3-nm bandpass filter (532FS03-50) is mounted to ensure that only elastic scattering is measured. The laser beam of a frequency doubled, 10 Hz, 7-ns pulsed Nd:YAG laser (Spectra-Physics Quanta-Ray PRO-250 set at 400 mJ per pulse) is sent through a half-wave plate to create optimal polarization for the Rayleigh signal strength. To create a thin, collimated light sheet in the area of interest, the laser beam passes three lenses. To ensure an almost even thickness of the sheet around the jet, a long focal length convex cylindrical lens (vertical-oriented, $f = 1000\,\text {mm}$) is placed at the focal distance from the axis of the injector. Subsequently, a horizontal Galilean beam expander pair expands and collaminates the 10-mm diameter laser beam into a 75-mm tall sheet using a negative cylindrical lens ($f = -20\,\text {mm}$) and a large positive cylindrical plano-convex lens ($f = 150\,\text {mm}$) placed at the sum of their focal lengths. Lastly, before entering the vessel through the slit windows, top and bottom cut-off knifes ensure that the laser sheet does not reflect on the injector tip or bottom of the window holder. The cut-off results in a 59-mm tall laser sheet in the region of interest. An overview of the optical setup to form the laser plane is shown in the right panel of Fig. 1.

Equation 1 shows that the scattered power increases linearly with Rayleigh cross-scattering efficiency, which depends drastically on the wavelength of incident light ($\sigma _i \propto \lambda ^{-4}$; Eq. 2). However, the reduced transmission through available camera UV lenses and the decrease in laser output under $\lambda = 532\,\text {nm}$ do not (yet) outweigh the increase in Rayleigh cross-section efficiency. Multiple flushes of the ambient gas and hydrogen are essential before making quantifiable measurements.

The fact that the incident laser light and the detection wavelength are identical potentially leads to interference by reflections off solid surfaces in the field of view and Mie scattering, especially by particles with a greater diameter than the incident wavelength (532 nm), without means to spectroscopically filter the images. Reflections are minimized by coating metal surfaces inside the vessel with a thin layer of soot, using anti-reflection-coated quartz windows, and directing a collimated laser sheet throughout the setup with window slits to block most reflections which originate from outside the chamber. Particle (Mie) scattering is minimized by the usage of 0.50-$\upmu \textrm{m}$ filters in the gas lines to block most of the particles entering the vessel. The remaining Mie scattering outliers are removed during post-processing using a Hampel filter (evaluating 10 points on either side, and three standard deviations). As both the ambient and jet scatter in a single measurement, beam steering due to density gradients between the jet and ambient can be corrected for in post processing. However, spurious reflections due to beam steering are inherent due to the turbulent nature of the jet and could not be remedied.

The mechanical shutter on the Pixis is relatively slow (5-ms opening and closing time) and the closing time between exposure and read-out is compensated for by the camera software. As the laser sheet is only present for 7 ns, the camera exposure starts 7 ms before the sheet passes, with an exposure time of 8 ms to ensure a fully open shutter when the light pulse arrives. This comes with the penalty of unwanted signal when there is no laser sheet, which is minimized by a dark working environment around the setup.

### Quantification

According to Eq. 3, the intensity value of a single pixel on the CCD-chip scales with both number density and the Rayleigh scattering cross-section of the species in the corresponding probe volume in object space. Note that the laser propagation direction is horizontal and that intensity variations are assumed to be only present in the vertical direction. The ratio between pixel values inside the jet region ($I_\textrm{r,jet}$) and to those in the (known) ambient upstream of the laser beam ($I_\textrm{r,a}$) equals [36]:

4 $$\begin{aligned} \frac{I_\textrm{r,jet}}{I_\textrm{r,a}} = \frac{\eta _\textrm{sys} I_0 }{\eta _\textrm{sys} I_0} \frac{N_\textrm{mix} \left( X_\textrm{f} \sigma _\textrm{f} + X_\textrm{a} \sigma _\textrm{a} \right) }{ N_{a,0} \sigma _\textrm{a} }, \end{aligned}$$

where $N_{a,0}$ is the number density of the unmixed ambient, outside of the jet.

This would lead to problems when both the number density (based on local pressure and temperature in a compressible medium) and the mole fractions are unknown. According to underexpanded jet theory [12, 13, 44], a highly underexpanded hydrogen jet can be assumed to be at chamber pressure ($p_\textrm{a}$) and stagnation temperature ($T^* = T_\textrm{f}$) from the Mach disk on. This allows for an isothermal approach at sufficient distance from the barrel shock and Mach disk as the fuel stagnation temperature is equal to the chamber temperature, where the number density is assumed constant in the far-field of the jet ($N_\textrm{mix} = N_{a,0}$). Equation 4 can then be rewritten for the mole fraction of the injected fuel, using $X_\textrm{f} + X_\textrm{a} = 1$:

5 $$\begin{aligned} X_\textrm{f} = \frac{\frac{I_\textrm{r,jet}}{I_\textrm{r,a}}-1}{\frac{\sigma _\textrm{f}}{\sigma _\textrm{a}}-1}. \end{aligned}$$

In an effort to investigate if local number density inside the jet is equal to the ambient, this work also includes a set of experiments where an argon jet was injected into an argon ambient (in subsection 3.2). As the species in the jet and ambient are equal ($\sigma _\textrm{f} = \sigma _\textrm{a}$), Eq. 4 can be rewritten for the number density ratio:

6 $$\begin{aligned} \frac{N_\textrm{mix}}{N_{a,0}} = \frac{I_\textrm{r,jet}}{I_\textrm{r,a}}. \end{aligned}$$

### Post-processing

**Fig. 2:**
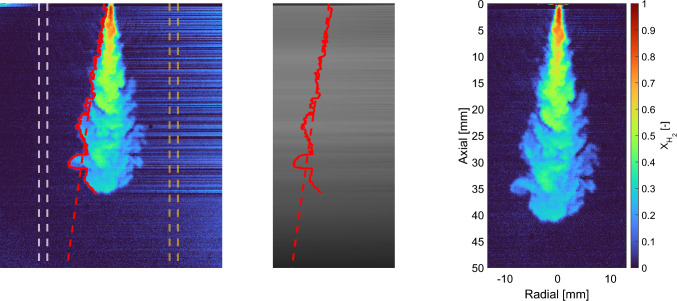
Outline of the post-processing of the Rayleigh scattering measurements. The left panel shows the mole fraction of a 10-MPa hydrogen jet into a 1-MPa nitrogen ambient, by obtaining $I_\mathrm {{r,a}}$ only upstream of the jet per row (dashed-white lines). The incident laser side of the jet is identified (in red), while the beam steering pattern is interpolated in that specific row until the dashed-ocher lines. The middle image shows the beam steering corrected background $I_\mathrm {{r,a}}$, including the earlier identified jet boundary from the left panel. The right panel shows the post-processed measurement, solving Eq. 5 for all pixels

The obtained raw measurements (*S*) are flat-field corrected to account for variations in the effective sensitivity of individual pixels. The flat-field corrected image (*C*) can be found with:

7 $$\begin{aligned} C =\frac{(S-F)\cdot m}{W-B}, \end{aligned}$$

where *m* is the averaged value of ($W-B$), *B* is the dark image (blocked lens) and *W* is a homogeneously illuminated white image. Image *W* was made by utilizing an exposure time of 100 ms to minimize the effect of the opening shutter in the image, which was visible at smaller exposure times. A so-called “flare frame” (*F*), consisting of a measurement on the evacuated ($p_\textrm{a}<{3}\,\text {kPa}$) vessel, is subtracted to largely remove contributions from stray light. Note that *W* and *B* are recorded in a separate experiment, not in the vessel. The flare frame *F* essentially corresponds to *B* plus contributions from stray light in the actual setup, which contributes $<1\% \,( X_\mathrm {H_2})$ to the uncertainty in the evaluated jet region. The images C contain the pixel values $I_\textrm{r,jet}$ of Eqs. 4–6; $I_\textrm{r,a}$ will be introduced below. As the remaining particles (outliers in intensity) are largely removed by the Hampel filter, the main concern for post-processing is correcting for the refractive index-gradient-induced beam steering, arising primarily from the jet’s periphery and (to a lesser extent) fluctuations inside the mixing jet. Beam steering is clearly visible to the right of the jet in the left panel of Fig. 2. However, inside the jet the beam steering pattern is unclear. For simplicity, it is assumed that the light only diverges from the red jet boundary to the dashed-ocher window downstream. The jet boundary is detected in a hydrogen mole fraction field (left panel) solved for Eq. 5, using a single ambient intensity per row ($I_\mathrm {{r,a}}$). This distribution per row is found by averaging ten columns (between dashed-white lines).

The background panel (containing $I_\mathrm {{r,a}}$, middle panel) cannot simply be obtained from an image without hydrogen injection but otherwise identical conditions, because of the jet-induced beam steering. Instead, as adapted from Idicheria and Pickett [36], it is created by a one-dimensional interpolation between the intensity values obtained outside the upstream jet boundary (red curve, left panel; note that “upstream" here refers to the laser beam direction) and the average value of the ten downstream columns of that specific row (dashed-ocher lines, left panel). As the boundary detection (10% $X_\mathrm {H_2}$ threshold) sometimes misses a row due to the end of jet penetration, particles or noise, a fit has been made through the boundary to increase robustness of the code and the most upstream (left, red) column is chosen to start the interpolation. The middle panel presents the resulting corrected background panel ${I_\textrm{r,a}}$ with the identical (red) jet contour and fit plotted inside, the map ends at the first ocher line of the left panel. The right panel of Fig. 2 shows the result when implementing $I_\mathrm {{r,a}}$ into Eq. 5. Most of the beam steering visible in the left panel is corrected for, but some noise ($<5\%$ of $X_\mathrm {H_2}$) remains outside of the jet boundaries.

## Results and discussion

### Ambient gas measurements

**Fig. 3:**
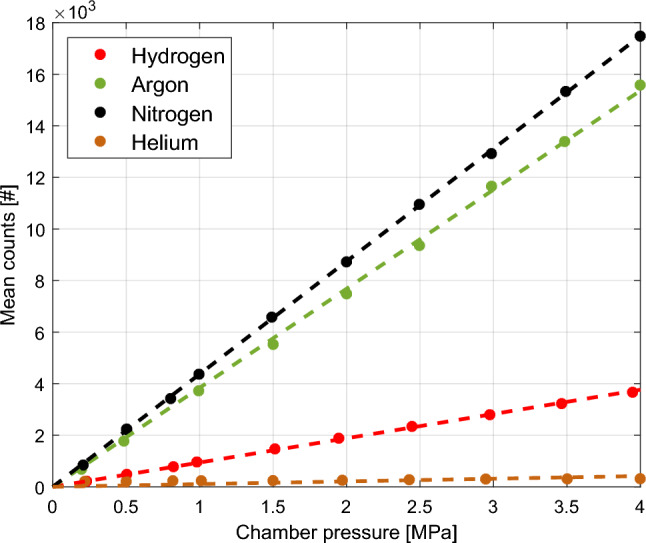
The mean intensity of the stencil placed in the middle of the setup and laser sheet for different (pure) gases and chamber pressures. The measurements are taken at constant temperature ($T_\textrm{a}= {291}\,\text {K}$) and averaged over 10 measurements

To verify that the experimental results correspond to the calculated Rayleigh cross sections in Table 1, the chamber is filled with pure gases at room temperature and an adjustable pressure between 0.2 and 4.0 MPa. According to Eq. 1 and the ideal gas law, the measured intensity values should scale with both pressure and the theoretical values of $\sigma _i$. The values in Fig. 3 correspond to the mean intensity of a region of $455\times 555$ pixels in the middle of the setup, where a big portion of the jet will be. The region is chosen in such a way that the borders of the laser sheet or sources of possible reflections like the injector tip, quartz windows, or setup inner walls, are at a decent distance. To account for shot-to-shot deviations in the laser’s output and residual particles (less than 5 visible in any image), every measurement point is averaged over 10 individual laser shots. The incident laser light power was measured and normalized between measurement sets.

Figure 3 shows relatively high pixel values for both the argon and nitrogen environments, while hydrogen yields lower intensity results, as expected from $\sigma _i$. Helium is also included, as it is an inert substitute for hydrogen in test environments, but its pixel values ($<100$ counts for the 1-MPa ambient) are clearly low compared to the other gases. This makes it impossible to use helium in a mixing experiment with the 16-bit camera, as any substantial pixel value for pure helium would mean immediate over-saturation in regions containing pure nitrogen or argon.

**Table 3:** Fitted slope and 95-% confidence bound, comparison between Rayleigh cross-section ratios for the pressurized pure gas environment experiments at constant room temperature ($T_\textrm{a}= 292\,\text {K}$) and theory from Table 1

Species	Slope, 95-% C.B.	Ratio to hydrogen	Theoretical ratio
–	# Counts/MPa	–	–
Argon	38.4 0.6	4.08 0.06	4.03
Nitrogen	43.7 0.2	4.65 0.02	4.61
Helium	0.94 0.2	0.10 0.02	0.061
Hydrogen	9.41 0.1	1	1

Linear fits were made (constrained to pass through the origin), where the slope returns the counts increase per bar. From Eq. 2 it follows that the ratio of these (mean) slopes for different gases should be equal to the corresponding ratio of scattering cross section. The results are summarized in Table 3. The error of approximately one percent can be a result of small differences in ambient temperature, residual shot-to-shot output differences and Mie scattering from particles. It should be noted that the mixing jet experiments are not prone to incident laser power variations as Eq. 5 is independent of $I_0$. Lastly, the helium ratio deviates substantially from the theoretical ratio (1.5 times higher), which can be explained by its low signal-to-noise and poor fit results. The helium counts are hardly above the dark noise counts due to helium’s small Rayleigh cross-section, where mainly remaining noise after the flare frame subtraction is mistaken for Rayleigh scattering counts by helium.

### Density ratio measurements

**Fig. 4:**
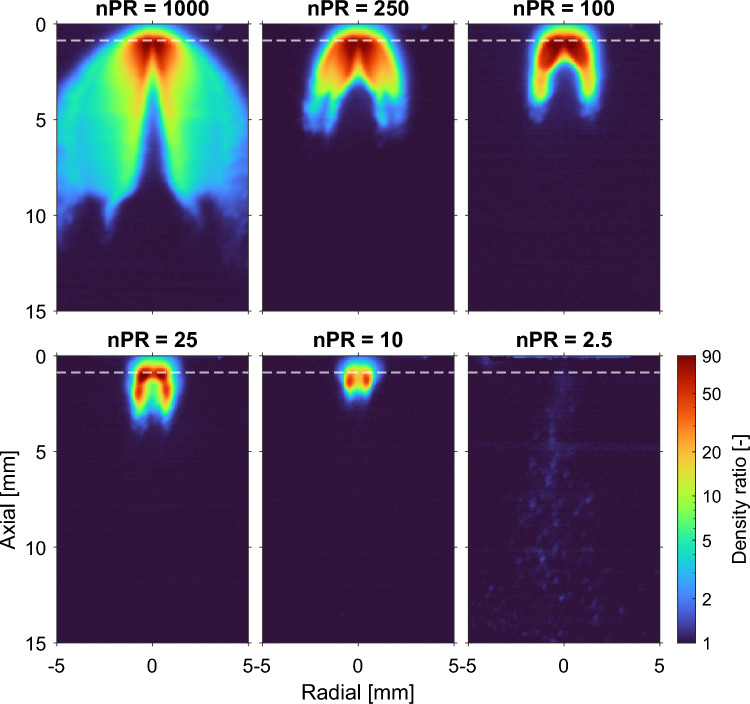
Near-nozzle measurements of the density ratio (that is, local Ar density in units of the ambient Ar density) for 10-MPa argon injections into argon ambients, averaged over 20 measurements using a logarithmic false-color scale. The dashed white line shows the upper border of the laser light sheet, 0.87 mm (10 pixels) downstream from the nozzle

A crucial assumption to arrive at Eq. 5 is that downstream of the Mach disk the jet density and temperature are essentially equal to the ambient. To evaluate this assumption, density ratio experiments were performed in an argon jet injected into an argon ambient, for nPR ranging from 2.5 to 1000. It is expected that the non-unity density ratio region depicts the barrel shock and ends at the Mach disk, as stated in theory [13, 44]. As the barrel shock and flow reach stability some time after the needle has fully opened, it was chosen to evaluate the density ratio 5 ms after the start of actuation. The results of these measurements are shown in Fig. 4, without the beam steering correction. The magnitude of the density ratio proves problematic against the saturation limit of a 16-bit camera system. By allowing relatively small pixel values outside the jet ($\approx 700$ counts), saturation can be minimized by operating the Nd:YAG laser at only 2 mJ per pulse. Despite the flat-field and flare correction, some resulting flare signal is still observed between the upper border of the laser sheet (dashed-white line) and the nozzle.

**Fig. 5:**
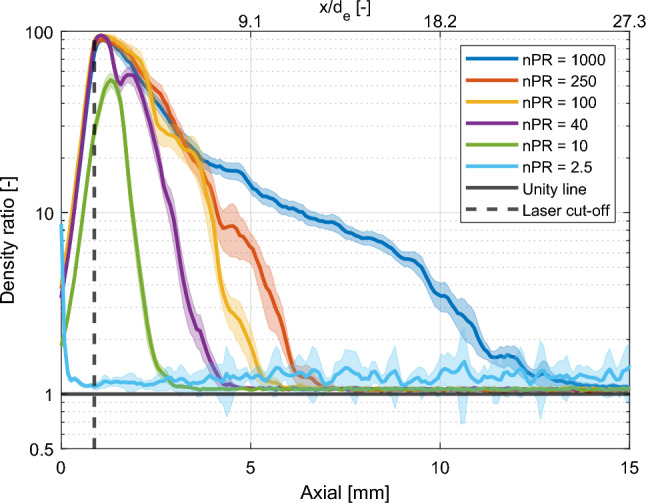
The density ratio of argon in the axial direction for different pressure ratios using a logarithmic scale. The values plotted (solid lines) correspond to the maximum density ratio in the radial direction, averaged over 20 measurements, including 95 percent confidence intervals (shading). The unity density ratio (solid black line) and the upper border of the laser (dashed black line) are also shown

Figure 5 shows the highest (radial) value plotted along the axial direction to indicate the distance for which non-unity values are expected. Even at these low power levels, saturation is achieved in a couple of pixels, capping the maximum density ratio ($\frac{2^{16}-1}{\sim 700} \approx 93$) for most pressure ratios. Due to the sheet distance from the nozzle and saturation in a couple of pixels, the exact magnitude at the highest density ratio is less reliable. Far away from the nozzle the density ratio is essentially unity. A small excess density in the jet region ($< 7\%$) is expected to be the result of residual background light. Note also that Fig. 5 corresponds to a “worst case scenario", emphasizing only the largest deviations. The nPR = 2.5 data were relatively strongly plagued by particulate scattering.

From Figs. 4 and 5 we conclude that the assumption of ambient number density is reasonable for $\textrm{nPR} \le 10$ from about 3 mm ($x/d_e = 5.5$) downstream of the nozzle. Where the assumption breaks down, this would give rise to relatively large Rayleigh scattering signal, and an underestimate of the local hydrogen mole fraction. The non-unity density ratio distance of the hydrogen jets is expected to be in the same order of magnitude as the classical results of Ewan and Moodie [12] show a major dependence on $d_\textrm{e}$, while a minor dependence is found on the ratio of specific heats.

### 10-MPa hydrogen into 1-MPa nitrogen

**Fig. 6:**
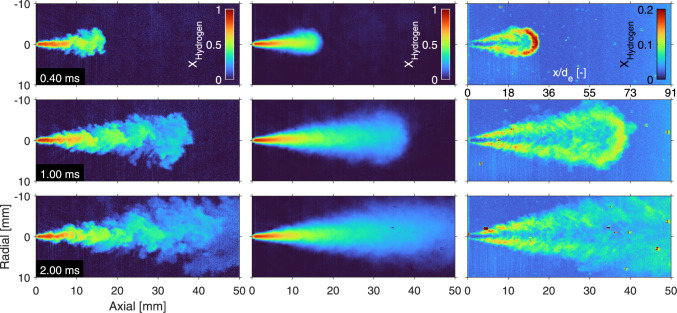
Evolution the 10-MPa hydrogen jet in a 1-MPa nitrogen ambient. The left panels show the instantaneous mole fraction fields measured by single laser shots at different timings aSOA, while the middle panels show an ensemble average over 20 separate jets. The right panels show the standard deviation of these 20 measurements in which particles in- and outside the jets, and beam-steering are still evident. Note that in reality the jets propagate from the top to the bottom of the vessel, under a small angle ($1.8^\circ$) to the perpendicular laser light sheet

Using the procedure and interpretation outlined previously, Fig. 6 shows some examples of the hydrogen density distribution of a 10-MPa hydrogen injection into a 1-MPa nitrogen ambient at three different times after SOA. The left panels show single injections, the middle panels show ensemble averages of 20 individual injections at the same timing, while the right panels show the standard deviation of these individual injections.

The tip of the jet is round and propagates in the axial direction with turbulent structures in the periphery that grow larger downstream. As these structures are not steady between individual jets and in time, the ensemble averages feature a relatively narrow jet core (in radial direction) as compared to the instantaneous results. The standard deviation panels highlight the unsteadiness in the periphery. The largest standard deviations are found in the tip of the 0.4-ms jet. At this timing, the relatively fuel-rich tip (when compared to later timings) differs the most between individual injections.

With all of the precautions, the number of particles over 20 individual measurements is low, but still visible. The standard deviation plots show a few particles (localized “dots" of large standard deviation) and slight residuals of the density-induced beam steering in the bottom. The Mie-scattering intensities are very high (compared to Rayleigh) and show very pronounced blooming on the chip of the Pixis camera, polluting multiple pixels in the process. Using Eq. 5, these very bright spots end up being interpreted as very high mole fractions of ambient gas instead of hydrogen. The post-processing creates an ambient intensity panel ($I_\textrm{r,a}$ in every pixel) that approximates the amount of beam steering downstream of the jet as well as the vertical laser light intensity distribution. However, remains of beam steering and slight differences in (vertical/axial) light distribution are still discernible in the standard deviation plots.

### Quasi-steady behavior

**Fig. 7:**
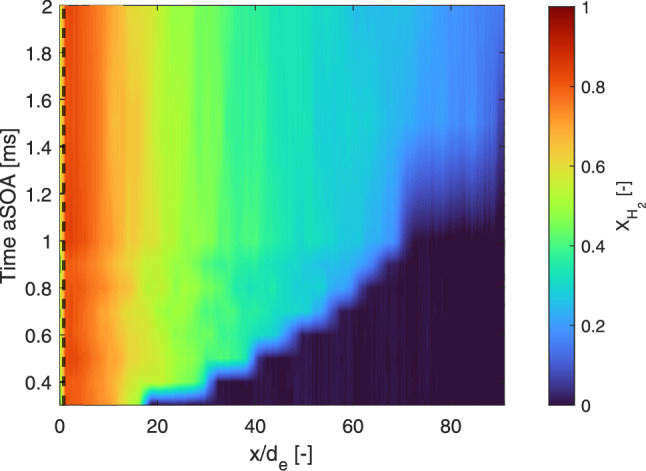
Interpolated time evolution of the hydrogen mole fraction on the normalized (axial) center line of the jet. The shown axial values are averaged over 20 single shots. The dashed black line shows the outer border of the laser sheet, at 0.87 mm from the exit hole

As mentioned in the previous section, the hydrogen mole fractions in the fuel-rich core of the jet (upstream of the tip) are highly reproducible, with only little variations between individual injections. To highlight the quasi-steady behavior, the axial evolution of the ensemble average is evaluated in Fig. 7. The (interpolated) time evolution of the center line shows an almost constant behavior in time, indicating such quasi-steady behavior in axial direction between the laser sheet border and just before the jet tip. Note that the staircase behavior of the tip is due to the limited time resolution, which is 0.1 ms before 1 ms aSOA and 0.50 ms thereafter.

**Fig. 8:**
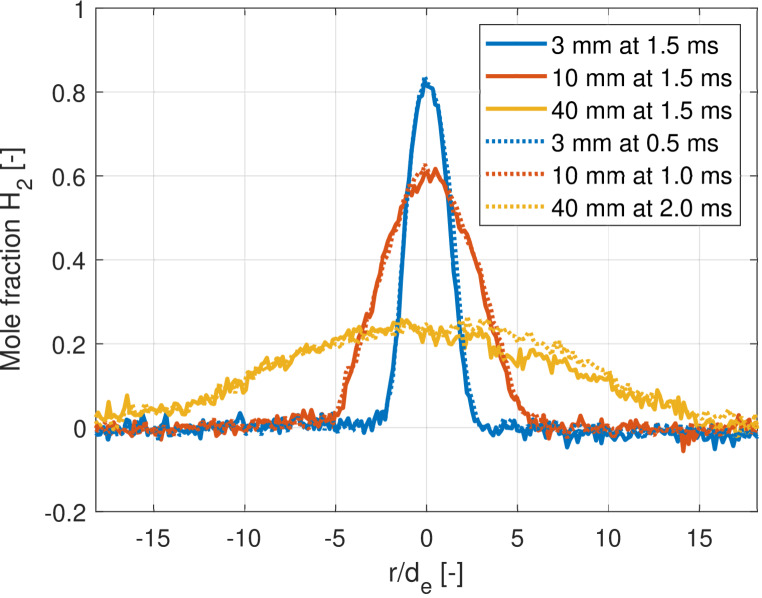
Time evolution of the radial mole fraction averaged over 20 single shots, evaluated at three axial distances. The solid line shows the jet at 1.5 ms aSOA, while the dotted lines show a variation of timings

To show full spatial quasi-steadiness of the jet in time, the radial profiles are evaluated at three distances (3, 10, and 40 mm) from the nozzle exit hole at various timings in Fig. 8. There is an almost perfect match between the timings, with a small deviation visible at 40 mm in the beam-steering side of the jet (positive radial values). The measured mole fraction is affected by residuals of beam steering and a particle at 2.4 mm. The mismatch of beam steering lowers the value at 1.5 ms by a couple of percentage points, while the single particle locally lowers the perceived mole fraction by more than five percent. Unfortunately, particle interference in elastic scattering is unavoidable when a mechanical injector is used, where metal-to-metal contact between the needle and seat is present.

### Self-similarity

Highly turbulent and axisymmetric jets show self-similar behavior, where their parameters scale with the axial distance from the nozzle exit hole. Due to non-ideal conditions in the nozzle (friction, non-isentropic expansion) and compressibility effects of high-pressure gases, one would not expect to find self-similarity close to the nozzle. In addition, a possible vortex head in the tip of the jet or differences in jet penetration between individual jets would also show different behavior to the rest of the jet.

**Fig. 9:**
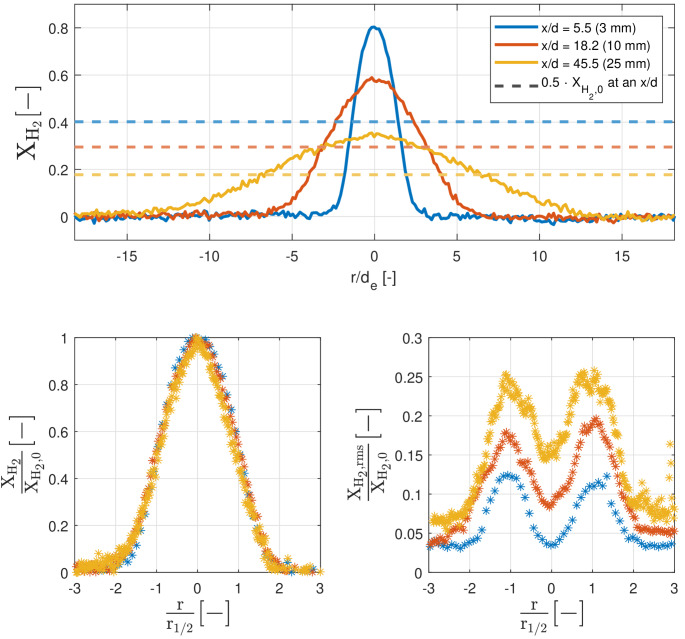
The top panel shows the quasi-steady radial mole fraction of the 10-MPa hydrogen injection into a 1-MPa nitrogen environment. The full width half maximum value is found at the intersection of the dashed lines and the according mole fraction at a specific axial distance. The bottom-left panel shows the normalized mole fraction against normalized radial distance of the jet. The bottom-right panel shows the normalized mean molar fraction fluctuations (root mean square) at the same axial distances

The upper panel of Fig. 9 shows the quasi-steady (Q-S) radial mole fraction of hydrogen (averaged over 110 measurements, ranging from 0.70--2.00 ms aSOA) at three different axial distances. Apart from some noise, the shapes are very symmetric over the center line. Half of the peak values at the center line ($X_\mathrm {H_2,0}$, dashed lines) are evaluated to obtain the Full Width Half Maximum (FWHM, $r_{1/2}$) of the radial profiles. $r_{1/2}$ is found by the intersection of the dashed $X_\mathrm {H_2,0}$ line and the radial profile. The bottom-left panel of Fig. 9 shows the normalized mole fraction of hydrogen (${X_\mathrm {H_2}}/{X_\mathrm {H_2,0}}$) versus the normalized radial distance. The normalized distance is obtained by dividing the radial axis by the FWHM (${r}/{r_{1/2}}$). The bottom-right panel shows the hydrogen molar fraction fluctuations ($X_\mathrm {H_2,rms}$), normalized in identical manner as the bottom-left panel.

The Q-S mole fractions show self-similar behavior as the normalized results from 3–25 mm from the nozzle show a similar trend as expected from incompressible axisymmetric wake [45–47] and round jet results [45, 48–51], but with minor differences visible between the different axial distances. The fact that self-similarity is found so close to the nozzle could be explained by the small exit diameter (0.55 mm). At more than five times the nozzle exit hole diameter, compressibility effects are expected to be negligible. Due to the expected density ratio, laser sheet border, and alignment of the sheet and hole, measurements closer to the nozzle are not possible. With a reduced signal-to-noise ratio for the case at a distance of 25 mm, small deviations are observed from the pattern followed by the 2- and 10-mm Q-S behavior. A slightly lower mean value is found near the center of the jet. The fluctuations (bottom-right panel) do not collapse after normalization with the mean centerline mole fraction, most probably due to a higher Reynolds number (expected $$\sim Re>10^6$$) than the axisymmetric wake and round jet, and the distance to the nozzle being much smaller than for the axisymmetric results, which are typically measured from $$\frac{x}{d_e}>30$$. The peak fluctuation values are found at $$ r=r_{1/2}$$, which is expected due to the maximum gradient of the mean at this radial location. This differs to the typical maximum variance found at the centerline in velocity measurements, as earlier found by Dowling and Dimotakis (1990) [48, 50, 51]. The non-zero fluctuation values when $$2<r/r_{1/2} <-2$$ are most certainly due to noise in the measurements, as the mean mole fraction reaches zero at these locations. Due to the normalization with the center line value, the non-zero fluctuations offset grows with increasing distance to the nozzle. These close-to-the-nozzle distances are of main interest for the temporally developing jets used in internal combustion engine applications, as ignition and combustion typically occur within this distance.

### Reduced injection pressure and equal nPR

As the jet formed by a high pressure ratio (nPR = 10) injection traverses the laser sheet in only 2 ms, the total volumetric flow is relatively small. For a pressure ratio of 2.5 the jet moves much slower and a longer injection is needed to fully traverse the light sheet, showing more particles inside a single measurement. Combined with the degradation (probably due to hydrogen embrittlement) of the (stainless) steel components over time and wear of the unlubricated needle on its seat, particle contamination becomes too problematic. Lowering the injection pressure only lowers the density inside the fuel system, but makes sure less particles flow out of the injector. As this allows the evaluation of longer injections (in subsection 3.8), it also allows for the evaluation of different fuel and chamber pressures at equal nPR. Using line-of-sight Schlieren and pressure-transducers in earlier work [16], the equal nPR jets were found to have identical geometry (jet angle and penetration), while the injected mass differed.

**Fig. 10:**
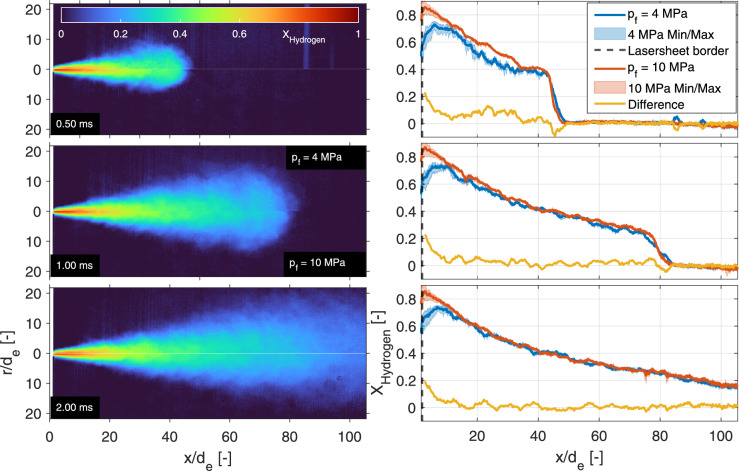
Left panels: half-jet comparison for both nPR = 10 conditions into the inert nitrogen ambient at three different timings. Both halves are taken from the incident laser side to minimize the effect of beam steering on the results. Right panels: Center line hydrogen mole fractions for both injection pressures at the same pressure ratio (nPR = 10) at three different timings. Solid lines represent the means at an axial location, while the shaded areas depict the minimum and maximum values within a 0.55-mm width. The yellow lines show the (interpolated) difference at that specific axial location (red−blue)

At an injection pressure of 4 MPa, these measurements are conducted using the same pressure ratio as above (nPR = 10). The left panels of Fig. 10 show the ensemble averaged hydrogen mole fraction of the 4 MPa injection pressure jet in the top half of each panel, and the 10-MPa injection pressure case in the bottom half, meaning that the incident laser side of the 4-MPa jet is mirrored over the jet axis, and both jets are corrected for the slight deviation from vertical propagation. The equal nPR jets in the left panels of Fig. 10 are essentially identical apart from the core in the 0.50-ms panel and the magnitude very close to the nozzle. In addition, the 0.4 MPa nitrogen ambient and jet contain a lower number density, which decreases the signal strength by a factor of 2.5, lowering the signal-to-noise ratio. The 0.50-ms panel also shows a distinct stripe in the lower injection pressure panel at $x/d_\textrm{e} = 85$, which is an artifact of a single particle in the $I_\textrm{r,a}$ creation window (on the red line or in the dashed ocher zone of Fig. 2). Where particles normally end up lowering the mole fraction locally, particles in the ambient creation windows decrease the post-processed ambient intensity ($I_\textrm{r,a}$), increasing all perceived hydrogen mole fraction in those specific rows. All measurements shown in this work were checked to exclude particles in the evaluation windows of the mentioned post-processing, which contaminate the results.

**Fig. 11:**
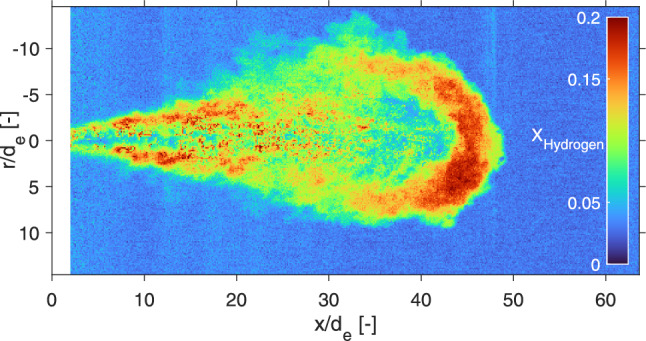
The standard deviation of 20 individual 4-MPa injections at $0.50\,{\text {ms}}$ aSOA. At these early timing the Rayleigh scattering image is heavily particle contaminated throughout the jet, lowering the apparent mole fraction

The center line mole fractions in the right panels of Fig. 10 tell a similar story, at 1.00 ms and 2.00 ms aSOA the jets look identical, apart from the first five to ten normalized distances close to the nozzle. A slight misalignment of the laser sheet to the heart of the nozzle hole probably misses the rich core close to the exit hole in the 4-MPa results. It makes sense for the jets to look identical, as both the number densities of the fuel and ambient scale linearly with the pressure. The 4 MPa at 0.50 ms panel shows something unexpected; from 15 to 30 normalized distances the mole fraction deviates substantially from all other center lines, with differences greater than 10 percent. The probable answer is found in the small particles that are present in later experiments, where it is expected that the needle and seat have worn. Figure 11 shows the standard deviation of the 20 individual injections, which show substantial particle contamination throughout the jet, as compared the top right panel in Fig. 6. Fine particles seem to mostly emanate from the needle, as most particles are found in the tip of the jet and during short timings between injector actuation and the laser pulse.

**Fig. 12:**
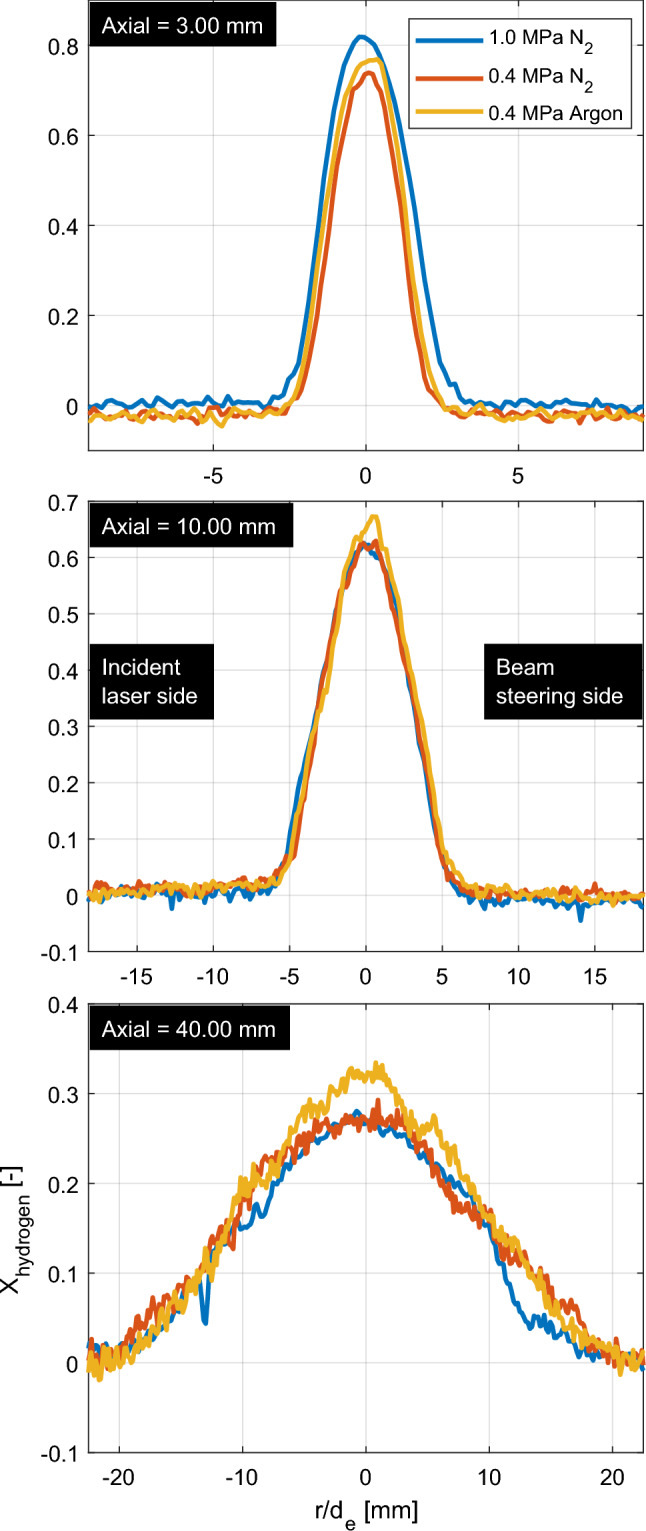
Radial mole fraction comparison at three axial distances of the nPR = 10 injections at 2.00 ms aSOA. Either the injection and ambient pressure (blue/red) or the species of the ambient gas (red/yellow) is varied

The radial results of the nPR = 10 ensemble averages at 2.00 ms aSOA in Fig. 12 are compared for both injection pressures into nitrogen (in blue and red) and for an 0.4-MPa argon ambient (yellow; discussed in more detail in subsection 3.7 below). For injections into nitrogen, the radial hydrogen mole fraction profiles at 10 mm from the nozzle are almost identical. The 0.4-MPa measurement contains more noise in the ambient, probably due to the lower number density. Similar to the axial profiles, the mole fractions are clearly lower at 3 mm from the nozzle in the 0.4-MPa injection into nitrogen. The diameter of the jet is slightly smaller, which is once more a reason to conclude that there has been a slight misalignment of the laser sheet to the heart of the exit hole. The background level (outside of the jet region) is slightly elevated for the 1-MPa jet, which differs due to small variations between the flare image (vacuum, no Rayleigh scattering) and the image containing the jet. These variations emanate from beam steering, as the jet is not present in the flare image, but they are small compared to the signal inside the jet region.

The overall mole fractions are lower in the 40-mm panel, while the width of the jet is substantially enlarged compared to the 3- and 10-mm panels. The lowered signal-to-noise becomes evident as well as the influence of particle contamination. A relatively big particle is present in the 1-MPa measurement, substantially lowering the mole fraction at the -12 normalized radial position. The zone around the 11–14 mm normalized radial distances appears lower for the 1-MPa results, which is expected to be due to individual jet variations of the turbulent jet at an increased distance from the nozzle.

### Nitrogen vs. Argon ambient

**Fig. 13:**
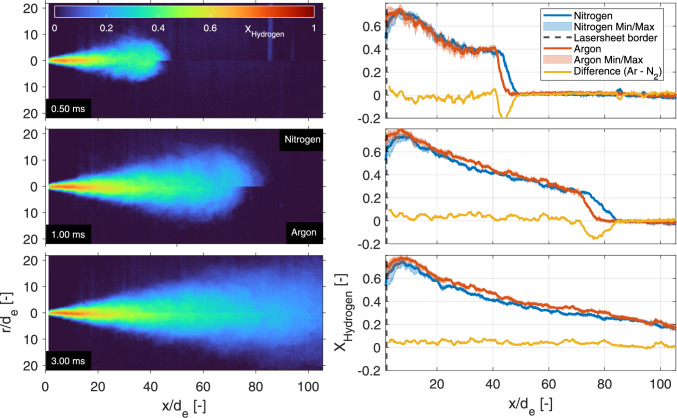
Left panels: half-jet comparison for both 4-MPa hydrogen injections (nPR = 10) into inert nitrogen (top side) and argon (bottom side) ambient gases at three different timings. Both halves are taken from the incident laser side to minimize the effect of beam steering on the results. Right panels: Center line comparison between both nitrogen (blue) and argon (red) ambient gases at the identical conditions ($p_\textrm{f} =$4 MPa, nPR = 10) at the three different timings

A comparison of mole fractions at the incident laser side between the 4-MPa hydrogen injections into 0.4-MPa nitrogen and argon environments is presented in the left panels of Fig. 13. Both cases are evaluated at equal conditions, no differences in laser sheet to injector alignment are present between the measurements. The results show a difference in jet tip penetration (as was found using high-speed Schlieren in [16]), while identical mass is injected. The mole fractions on the center line are presented in the right panels of Fig. 13 and radial results previously shown in Fig. 12 (red/yellow curves). In Fig. 13, the centerline differences in mole fraction to compensate for the difference in jet penetration are small ($<5\%$), but visible in the centerline plots (Fig. 13). Again, the 0.50-ms panel is heavily particle contaminated in all individual jets (with similar standard deviation as in Fig. 11), decreasing the measured ensemble average mole fraction in both ambients.

Interestingly, when evaluating the radial profiles in Fig. 12, mainly the core appears slightly richer, while the width of the jets seems identical. The hydrogen mole fraction is always higher for an identical pressure and temperature argon ambient in the core of the jet region, indicating that the core of the jet mixes better in a nitrogen environment. The width (and thus angle) of the jet in the radial profiles is largely unaffected by the ambient species.

### nPR = 2.5

**Fig. 14:**
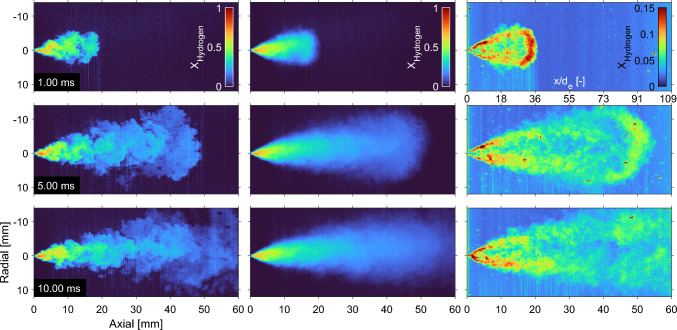
Evolution of the 4-MPa hydrogen jet in a 1.6-MPa argon ambient. The left panels show the hydrogen mole fraction of single shots at different timings, while the middle panels show an ensemble average over 20 jets. The right panels show the standard deviation of these 20 measurements, where particles and beam-steering are visible throughout the measurements

Hydrogen is typically stored at 35 to 70 MPa and as further compression of hydrogen is relatively energy intense [5], very high pressure ratios are not expected compared to the typical in-cylinder pressure of combustion engines. To stay above the choked flow limit for a good prediction of mass flow, a fuel line to chamber pressure ratio of 2.5 was identified to be a reasonable lower pressure ratio for future combustion engines. Lowering the pressure ratio decreases jet penetration substantially and calls for long (10 ms) injections to reach the end of the laser sheet. Figure 14 presents individual injections (left panels), ensemble averages (middle panels) and the standard deviation (right panels) of 20 injections of 4 MPa hydrogen into 1.6 MPa argon. As the choked mass flow remains identical to the $p_\textrm{f} = {4}\,\text {MPa}$, $\textrm{nPR} = 10$ cases, but as jet penetration decreases, a difference in geometry and mole fraction throughout the jet is expected.

Indeed, the jet becomes much wider and has a lower overall mole fraction throughout the ensemble-averaged jet area after 10 ms when compared to the jet after 2 ms in Fig. 6. As the overall number density in the chamber is now four times higher than for a 0.4 MPa ambient, a direct comparison of mixing becomes challenging. However, at the same time interval (1 ms aSOA, and thus same injected mass), the nPR = 2.5 jet is wider, higher hydrogen mole fractions are measured throughout the jet, and the jet remains relatively close to the nozzle, compared to the nPR = 10 case.

In contrast, the near field shows lower mole fractions than found in the nPR = 10 jet. The turbulent structures close to the nozzle are already wider in the nPR = 2.5 case, but show similar standard deviation ($\sim 0.15$) as the narrower structures visible in the nPR = 10 case. The beam-steering pattern outside of the jet in the standard deviation panels also appear similar (standard deviation of $\sim 0.05$). As the ambient number density is higher, the signal-to-noise ratio increases relative to the other conditions. Less noise is visible in the background of the single-shot images and there is an overall decrease in the standard deviation outside of the mixing jet. In addition, the jet is less symmetric over the axial direction than at nPR = 10. The background (ambient) shows a structured standard deviation, where shot-to-shot differences in the laser sheet distribution become evident as a pattern in axial direction. As injections are three times as long, more particles are present throughout the measurements.

## Conclusions

Instantaneous mole fraction distributions of high-pressure hydrogen injections into either argon or nitrogen ambients under choked flow conditions through a 0.55-mm diameter nozzle were quantified by Rayleigh scattering using a non-intensified CCD camera and a frequency-doubled Nd:YAG 10-Hz laser system. The sensitivity of the setup was such that interpretable Rayleigh scattering measurements could be performed using single laser pulses (<10 ns duration), allowing to essentially freeze the flow in time. Relative Rayleigh scattering cross sections for various pure gases were found to be consistent with literature values ($\approx 1 \%$ deviation), validating the measurement and post-processing protocols. Measures were taken to minimize the number of dust particles in the images.

A major assumption in the quantification of hydrogen mole fractions in the mixing jet is the assumption of the equal number density between jet and ambient (“unity density ratio assumption"). The assumption was validated using high-pressure argon jets injected into argon, for which the scattering cross section is spatially uniform. The near-field zone of the jet (dominated by compressibility effects) was found to extend at most until 3 mm downstream of the nozzle (z/d = 5.5) for nPR<10. Beyond that, the unity density ratio approximation was found to hold for all conditions used. These conditions will not be equal for hydrogen being injected into the ambient gas, but are expected to be in the same order of magnitude.

The time evolution of the 10-MPa hydrogen injections into a 1-MPa nitrogen environment shows the behavior expected from theory; the ensemble-averaged jets were found to be symmetric, showing both axially and radially quasi-steady behavior in time as well as self-similar behavior of the hydrogen mole fraction field. However, the normalized hydrogen mole fraction fluctuations do not collapse in a similar manner.

An equal pressure ratio jet (4 MPa hydrogen into 0.4 MPa nitrogen) was found to exhibit identical behavior for both fuel pressures. The hydrogen injections into equal pressure argon and nitrogen environments showed similar results, but jet tip penetration in argon was found to always be slower. This results in higher mole fractions in the richer core region, while the width remains identical.

The pressure ratio of ten is quite high for direct injection of hydrogen into a pressurized combustion (engine) chamber, so a pressure ratio of 2.5 was considered as the lower limit. Jet tip penetration was found to slow down by a factor of about three as compared to injections at the same stagnation pressure, but with nPR = 10. At an identical timing (1 ms aSOA) the jet is much shorter, wider, less symmetrical and it contains higher mole fractions throughout, apart from the near nozzle region. In addition, more fine particles are present due to the longer injection duration. It is expected that these –probably due to hydrogen embrittlement– emanate from the needle-to-seat contact as the injector closes, because the injector does not contain any lubrication.

The quantitative, spatially resolved mole fraction distributions are a direct measurement of the hydrogen jet mixing with the pressurized environment at room temperature, which is a major improvement compared to line-of-sight integrated Schlieren measurements performed in the past. The mole fraction fields are expected to be valuable for CFD validation and modeling in general to improve the understanding of high-pressure hydrogen injections for future hydrogen internal combustion engines and the pursued Argon Power Cycle. In addition, the quasi-steady behavior of the ensemble averages allows less sensitive measurement techniques such as spontaneous Raman scattering to address the mean behavior of parameters which can not be measured using Rayleigh scattering, such as temperature and mole fraction in the near nozzle region.

## Supplementary Information

Below is the link to the electronic supplementary material.

Supplementary file 1 (stl 143 KB)

## Data Availability

The inner-nozzle geometry (.stl) is supplemented to aid modeling efforts, making numerical validations possible. Data presented in graphs can be shared upon request.

## List of symbols

$\gamma$: Specific heat ratio
$I_\mathrm{s}$: Intensity from Rayleigh scattering # counts
$I_\mathrm{0}$: Incident (laser) intensity # counts
V: Probe volume ($${m}^3$$)
L: Distance to the detector (m)
$$R_\mathrm{s}$$: Scattering efficiency ($$\textrm{m}^{-1}$$)
N: Number of molecules
$\sigma _i$: Scattering cross section of i ($\textrm{cm}^2$)
$$\lambda $$: Wavelength (nm)
$n_i$: Refractive index of i
$N_i$: Molecular number density ($\textrm{m}^{-3}$)
$\rho _v$: Depolarization ratio of i
A: Surface area ($$\textrm{m}^2$$)
$P_\mathrm{det}$: Incident power on detector # counts ($\textrm{m}^2$)
$\Omega$: Solid angle sr
$\eta _\mathrm{s}$: Detection system efficiency
$I_\mathrm{r}$: Intensity per pixel # counts/pixel size
$d_\textrm{e}$: Nozzle exit hole diameter (mm)
$X_i$: Mole fraction of i
$$n_\mathrm{a}$$: Ambient species
$p_i$: Absolute pressure of i (MPa)
$$\dot{m}$$: Mass flow ($$\mathrm {{mg}/{ms}}$$)
$$C_\textrm{d}$$: Discharge coefficient
$t_\textrm{inj}$: Injection duration (actuation) (ms)
t: Time (ms)
nPR: Nozzle pressure ratio
f: Focal length (m)
$$T_i$$: Temperature of i (K)
$X_\mathrm {H_2,0}$: Centerline value of hydrogen mole fraction
$r_{1/2}$: FWHM radial distance (mm)
$X_\mathrm {H_2,rms}$: Fluctuations (root mean square) of hydrogen mole fraction
